# Unmanned Aerial Vehicle-Based Phenotyping Using Morphometric and Spectral Analysis Can Quantify Responses of Wild Tomato Plants to Salinity Stress

**DOI:** 10.3389/fpls.2019.00370

**Published:** 2019-03-29

**Authors:** Kasper Johansen, Mitchell J. L. Morton, Yoann M. Malbeteau, Bruno Aragon, Samir K. Al-Mashharawi, Matteo G. Ziliani, Yoseline Angel, Gabriele M. Fiene, Sónia S. C. Negrão, Magdi A. A. Mousa, Mark A. Tester, Matthew F. McCabe

**Affiliations:** ^1^Hydrology, Agriculture and Land Observation, Water Desalination and Reuse Center, King Abdullah University of Science and Technology, Thuwal, Saudi Arabia; ^2^Center for Desert Agriculture, The Salt Lab, King Abdullah University of Science and Technology, Thuwal, Saudi Arabia; ^3^School of Biology and Environmental Science, University College Dublin, Belfield, Ireland; ^4^Department of Arid Land Agriculture, Faculty of Meteorology, Environment and Arid Land Agriculture, King Abdulaziz University, Jeddah, Saudi Arabia; ^5^Department of Vegetables, Faculty of Agriculture, Assiut University, Assiut, Egypt

**Keywords:** UAV, imagery, phenotyping, wild tomato, *Solanum pimpinellifolium*, salt tolerance, growth, yield

## Abstract

With salt stress presenting a major threat to global food production, attention has turned to the identification and breeding of crop cultivars with improved salt tolerance. For instance, some accessions of wild species with higher salt tolerance than commercial varieties are being investigated for their potential to expand food production into marginal areas or to use brackish waters for irrigation. However, assessment of individual plant responses to salt stress in field trials is time-consuming, limiting, for example, longitudinal assessment of large numbers of plants. Developments in Unmanned Aerial Vehicle (UAV) sensing technologies provide a means for extensive, repeated and consistent phenotyping and have significant advantages over standard approaches. In this study, 199 accessions of the wild tomato species, *Solanum pimpinellifolium*, were evaluated through a field assessment of 600 control and 600 salt-treated plants. UAV imagery was used to: (1) delineate tomato plants from a time-series of eight RGB and two multi-spectral datasets, using an automated object-based image analysis approach; (2) assess four traits, i.e., plant area, growth rates, condition and Plant Projective Cover (PPC) over the growing season; and (3) use the mapped traits to identify the best-performing accessions in terms of yield and salt tolerance. For the first five campaigns, >99% of all tomato plants were automatically detected. The omission rate increased to 2–5% for the last three campaigns because of the presence of dead and senescent plants. Salt-treated plants exhibited a significantly smaller plant area (average control and salt-treated plant areas of 0.55 and 0.29 m^2^, respectively), maximum growth rate (daily maximum growth rate of control and salt-treated plant of 0.034 and 0.013 m^2^, respectively) and PPC (5–16% difference) relative to control plants. Using mapped plant condition, area, growth rate and PPC, we show that it was possible to identify eight out of the top 10 highest yielding accessions and that only five accessions produced high yield under both treatments. Apart from showcasing multi-temporal UAV-based phenotyping capabilities for the assessment of plant performance, this research has implications for agronomic studies of plant salt tolerance and for optimizing agricultural production under saline conditions.

## Introduction

Approximately 20% (45 million ha) of irrigated land is salt-affected (Machado and Serralheiro, 2017). Salt stress, caused by either saline or sodic soils, represents a major threat to global food production (Rao et al., 2013), with estimates of up to $30 billion in agricultural losses annually (Qadir et al., 2014). The problem is particularly acute in arid and semiarid environments, where irrigation associated with insufficient drainage of water from the sub-soil causes saline waters to rise into the root zone (Pitman and Lauchli, 2002). Salinity also occurs in irrigated soil because of the accumulations of soluble salts introduced via the continuous use of irrigation waters containing medium to high quantities of dissolved salts (Al-Hassoun, 2007; Ferchichi et al., 2018). Ultimately, excess salts cause a water deficit in plants due to osmotic stress and lead to the accumulation of sodium ions in plant shoots where they disrupt key biochemical processes (Zhang and Blumwald, 2001; Rao et al., 2013), resulting in yield losses. With estimates of global crop production needing to increase by more than 60% by 2050 (United Nations World Water Assessment Programme [WWAP], 2014; Senthilnath et al., 2016), breeding crops with improved salt tolerance represents a research priority (Munns and Tester, 2008; Messerer et al., 2018).

The commercial tomato is one of the world’s major horticultural crops, with a global annual production of approximately 178 million tons (FAOSTAT, 2016). Tomato varieties generally tolerate salinity levels up to 2,500 μS/cm, but above this level, the quality and yield often declines. To overcome yield losses due to salinity, the use of salt-tolerant wild tomato species as a genetic resource for improving commercial varieties has been explored. Indeed, some accessions of the wild tomato species *S. pimpinellifolium*, have shown traits of increased salt tolerance, representing a potential candidate for breeding (Zhang and Blumwald, 2001; Rao et al., 2013; Razali et al., 2018). As it is closely related to *S. lycopersicum*, *S. pimpinellifolium* has been used as a donor for many commercially important tomato traits (Zuriaga et al., 2009; Rao et al., 2013; Razali et al., 2018). *S. pimpinellifolium* is native to Peru and Ecuador, where it is adapted to diverse environmental conditions, ranging from coastal desert climates to humid and foggy conditions at higher altitudes (Zuriaga et al., 2009; Rao et al., 2012). With this diversity of environmental adaptions, some *S. pimpinellifolium* accessions might be suited to arid environments with saline soils elsewhere in the world.

Field trials are used to assess plant responses to soil conditions, fertilizers, diseases, abiotic stressors (e.g., heat, water, nutrients, wind, salinity) and many other growth factors in agriculturally and economically relevant settings (Singh et al., 2016). Plant phenotyping, i.e., the assessment of a plant’s observable characteristics and traits, such as its architecture, and biochemical and biophysical properties, is performed in order to identify key determinants of growth and yield (Yang et al., 2017). Effective field-based phenotyping is still considered a bottleneck to improve efficiency in breeding programs (Singh et al., 2016). While field trials are an effective setup for assessing plant traits and responses to different types of abiotic stress factors, phenotyping large numbers of plants in the field is often time-consuming, labor-intensive and subjective, especially for collection of time-series data that demand repetitive collection procedures (Sugiura et al., 2015; Holman et al., 2016). Given that abiotic stresses adversely affect photosynthesis and the growth of stems, leaves, and roots and, consequently, yield and fruit quality, there is potential for remote sensing technologies to measure these manifested characteristics in a more efficient and consistent manner. Indeed, a number of remote sensing approaches have already been developed for rapid and non-destructive assessment of responses to biotic and abiotic stress in tomato plants (Li et al., 2014).

Zhang et al. (2002, 2003, 2005) used field spectroscopy, airborne hyper-spectral and airborne multi-spectral imagery to map tomato plants with late blight infection, finding that only those plants with middle to late stages of infection could be mapped. Their assessment, however, focused on patches in a field rather than individual tomato plants, because of insufficient spatial resolution of the airborne imagery available. More recent developments in the use of Unmanned Aerial Vehicles (UAV) provide the capability to obtain imagery with a much higher spatial and temporal resolution, allowing individual plants and their properties to be clearly differentiated (Shi et al., 2016; Patrick and Li, 2017; Jung et al., 2018). Candiago et al. (2015) used a UAV-mounted Tetracam to calculate three different vegetation indices for assessment of tomato plants. However, they did not invert the indices to estimate biophysical or biochemical properties, and their assessment was not carried out at the individual plant level. Therefore, these results may have been affected by exposed bare ground in between plants. Senthilnath et al. (2016) used two UAV-derived images to map individual tomato fruits of each plant, testing different segmentation and classification approaches. They found this task to be difficult, as many fruits were hidden by the leaves and stalks. Moeckel et al. (2018) estimated crop height and biomass of eggplant, tomato and cabbage plants from UAV-based Red-Green-Blue (RGB) imagery and Structure-from-Motion and found a good correlation with manual field observations. Enciso et al. (2019) used RGB and multi-spectral UAV imagery collected during the growing season of eight difference tomato varieties to estimate plant height, canopy cover and NDVI, and found that height could be accurately estimated and that canopy cover was highly correlated with field-based LAI measurements. However, this assessment was performed at the plot and not individual plant level.

With an increasing number of studies focusing on UAV-based high-throughput phenotyping of agricultural crops, methods that might be appropriate for studies of tomato plants are also increasing. For instance, Makanza et al. (2018) used UAV-based RGB imagery to assess crop cover and canopy senescence in a maize field trial and found that the UAV imagery-derived plant traits showed moderately high heritability values for both traits. Holman et al. (2016) used multi-temporal UAV-based RGB imagery to measure wheat plant height, and found the image time-series useful for estimating growth rates in relation to fertilizer rates. Tattaris et al. (2016) obtained statistically significant correlations between UAV-based thermal and multi-spectral image-derived canopy temperature and Normalized Difference Vegetation Index (NDVI) values in relation to biomass and yield of wheat. Patrick and Li (2017) used UAV-based RGB imagery to successfully measure structural parameters (height, extent, canopy area, crown diameter and width) of 25 blueberry bushes. Many other recent examples of UAV-based applications for high-throughput phenotyping exist, e.g., for responses to drought and nitrogen deficiency in dry bean (Sankaran et al., 2018), vigor of different barley genotypes (Di Gennaro et al., 2018), drought adaptive traits in durum wheat (Condorelli et al., 2018), sorghum breeding for estimation of plant height (Watanabe et al., 2017; Hu et al., 2018), black poplar response to drought (Ludovisi et al., 2017), and for estimating the intra-field crop height variability at commercial farm scales (Ziliani et al., 2018). The reviewed literature on UAV-based high-throughput phenotyping demonstrates the capability of multi-temporal RGB and multi-spectral imagery for assessing growth rates, architectural parameters and plant cover under abiotic stress and control conditions.

While there has been a recent increase in agronomic research using UAV-based high-throughput phenotyping of agricultural crops (Shi et al., 2016), a survey of the literature indicates that our study is the first one to interrogate individual tomato plants in detail and demonstrate the utility of phenotyping tomato plant traits toward assessing yield and salt tolerance. The aim of this research was to develop and demonstrate a UAV-based method for effective assessment of phenotypic characteristics and salt tolerance of 199 wild tomato (*S. pimpinellifolium*) accessions in a field trial. Specific objectives were to: (1) automatically delineate tomato plants from a time-series of eight RGB and two multi-spectral UAV image datasets, using an automated object-based image analysis approach; (2) assess plant area, growth rates (defined as daily changes of plant area), condition and Plant Projective Cover (PPC, i.e., the vertically projected leaves, suckers, flowers, stem and fruit) over the growth season; and (3) identify the best-performing accessions in relation to yield and salt tolerance. Additional research contributions include using UAV-based image time-series analysis for multi-temporal plant trait analysis and determining their relations to salt tolerance and performance in terms of yield. The developed UAV-based methods provide the foundation to support more detailed plant phenotyping using remotely sensed mapping and monitoring of plant area, growth rates, biophysical and biochemical traits and health status of different tomato plant varieties at the individual plant and even sub-plant level.

## Materials and Methods

In this study, four phenotypic traits were mapped from multi-temporal UAV imagery, including plant area, growth rate, condition and PPC. While not previously explored for tomato plants using UAV data, these traits were selected due to their previous correlation with plant yield in other crop types (Peñuelas and Filella, 1998; Holman et al., 2016; Lootens et al., 2016; Jung et al., 2018; Makanza et al., 2018). To assess these traits at the individual plant level, an object-based image analysis approach was used as this technique has proven most suited for imagery with high spatial resolution, where mapped features may consist of hundreds or thousands of pixels (Blaschke, 2010).

### Study Area and Experimental Design

**FIGURE 1:**
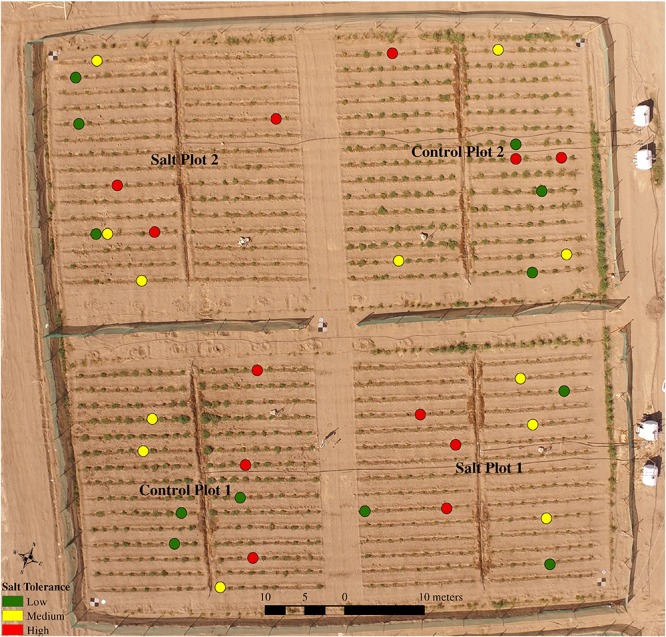
Overview of study site at King Abdulaziz University Agricultural Research Station in Hada Al-Sham on the January 14, 2018, showing the four plots and four water tanks for irrigation as well as 36 plants with low, medium and high salt tolerance selected for focused field analysis.

The study area was located at the King Abdulaziz University Agricultural Research Station in Hada Al-Sham (21°47′48″N, 39°43′35″E), approximately 60 km east of Jeddah, Saudi Arabia. The site is located in a tropical arid climate that receives less than 100 mm of rainfall annually, and has a predominantly sandy loam soil type. An area of 75 m × 75 m was established for the experiment, comprising four plots of approximately 30 m × 30 m per plot, with each containing 15 rows of 20 tomato plants (Figure 1). A total of 1,200 tomato plants were planted, consisting of 200 genotypes that included 199 *S. pimpinellifolium* accessions (Supplementary Table S1) and one *S. lycopersicum* accession (the commercial tomato, Heinz 1706). The 199 *S. pimpinellifolium* accessions were originally collected in the 1950s and 1960s from different sites in Peru and Ecuador. The seeds for these accessions were obtained from the Tomato Genetics Resource Center at the University of California Davis and propagated at King Abdullah University of Science and Technology (KAUST) to generate a stock of fresh seeds for use in this experiment.

All tomato plants were sown between October 1–2, 2017 at a greenhouse nursery at KAUST and transplanted, following a randomized design, 1 month later between November 1–2, 2017 into two control and two salt-treated plots. Each of the 200 accessions had three replicates in each of the two treatments. The two control plots were irrigated solely with low salinity water (approximately 27 mM NaCl, 900–1000 ppm), while the two salt-treated plots were irrigated with water of increasingly saline concentration. In those plots, the salt concentrations ranged from the original low salinity water to 127 mM NaCl (4500 ppm) from November 14 2017, 197 mM NaCl (7000 ppm) from December 4, 225 mM NaCl (8000 ppm) from December 10, 254 mM NaCl (9000 ppm) from December 18, and 183 mM NaCl (6500 ppm) from January 12 2018 until the time of harvest (between January 16–22). Drip irrigation occurred twice daily, first in the morning around sunrise and then in the evening after sunset, with both lasting for 10 min in the first week, 15 min from the second week (November 9), and 30 min from the eighth week (December 17) until harvest, in line with the increasing water requirements of growing plants. To ensure pure vegetation signals from the plots, any weeds were removed manually before each of the UAV flights. Maximum day and minimum night temperatures ranged from 27 to 37°C and 12 to 24°C, respectively, with a mean temperature of 25.67°C during the growing season. No rainfall was recorded, but several sandstorms occurred during the growing season, including on December 8 and 16 2017, on January 4, and between January 8–10 2018. Workers washed the plants with non-saline water and cleaned the plots after each event to prevent reflectance attenuation of the plants.

### Field Data for Geometric and Radiometric UAV Image Calibration

Eight field campaigns were undertaken on November 9, 16, 23, and 30 and on December 6 and 20 2017, together with January 7 and 14 2018. To support the UAV-based imagery, a range of *in situ* data were collected concurrently, including ground control points (GCPs) for geometric correction of the UAV, spectrometer measurements of radiometric calibration panels, measurements of plant dimensions, visual assessment of plant condition, and ground-based plant photography for PPC measurements. Five GCPs were installed and measured on the November 2 planting date using a Leica GS10 base station with an AS10 antenna and a Leica GD15 smart antenna as a rover (Leica Geosystems, St Gallen, Switzerland). A single GCP was placed in the center of the field site, and another at each of the four corners of the study domain. All raw data from the base station and rover were post-processed using Leica Geo Office (Leica Geosystems, St Gallen, Switzerland). Six radiometric calibration panels were produced using oak plywood boards painted with three coats of matte paint in white, four shades of gray, and also in black (Johansen et al., 2018). The reflectance values of the six targets were measured with an ASD FieldSpec4 spectrometer (Malvern Panalytical, Malvern, United Kingdom) and confirmed to be near Lambertian. The root mean square error (RMSE) of reflectance (scaled from 0 to 100%) was 0.17%, between 450 and 850 nm, corresponding to the spectral range of the collected UAV RGB and multi-spectral imagery. The RMSE was based on spectrometer measurements obtained at 13 different elevation and azimuth angles, i.e., at nadir and at approximately 15, 30 and 45° off-nadir angles viewed from north, south, east and west, as suggested by Johansen et al. (2018).

### Field-Based Plant Phenotypic Measurements

Measurements of morphometric features, condition and PPC were undertaken on January 7, 2018 for 36 selected tomato plants in the field. These tomato plants belonged to six accessions, deemed to have high (2 accessions), medium (2 accessions) and low (2 accessions) tolerance to saline irrigation (based on results from an earlier field trial). Each of the six accessions had six replicates (i.e., 36 plants in total), with three replicates of each of the six accessions (i.e., 18 plants) planted in both the control and salt-treated plots (for a total of nine plants in each of the four sub-plots) (Figure 1). It should be noted that because of the uneven number, i.e., three, of control and salt-treated replicates, these were split unequally between plots, i.e., one salt-treated replicate in one salt plot and two salt-treated replicates in the other salt plot. The selection of nine plants per plot used for focused analysis was a compromise between the number of plants that were practically feasible to assess within a day and obtaining sufficient information on plant biophysical and biochemical characteristics every second week during the growing season for calibration/validation purposes.

**FIGURE 2:**
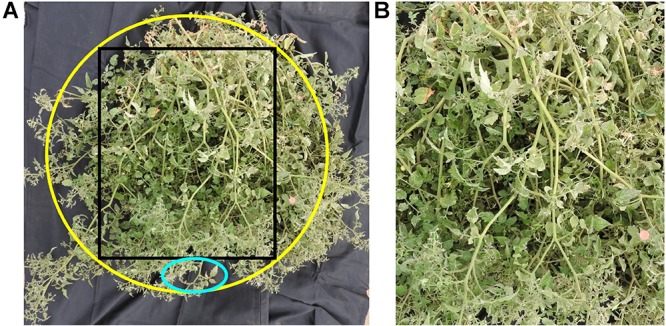
**(A)** Tomato plant with black material as background. The yellow ellipse shows the approximate perimeter of the plant, while the black square shows the part of the photo analyzed in **(B)**. The cyan ellipse shows how areas with no plant material were excluded to correspond with the UAV-based plant delineation results. In this particular example, the studied plant had a PPC of 91.31%.

While the area of each plant was difficult to estimate in the field, ground-based measurements of the length and width of the tomato plants provided quantitative measures that could be directly compared against the UAV imagery. The length of each of the 36 plants were measured along the plants’ longest axis, with the longest width measured perpendicular to this axis. To measure the PPC for the same 36 plants, one representative photo was taken at midday on January 7 from a position vertically above each tomato plant, after black material had been placed underneath the plant canopy. The black background more easily enabled the separation of plant material and gaps within the canopy. Prior to analysis, each photo was cropped to exclude any edge effects from irregularly shaped tomato plants and to enable subsequent analysis to correspond to the UAV-based plant delineation. To be consistent, an ellipse was drawn along the approximate perimeter of the tomato plants and a rectangle was placed within the ellipse and used for cropping the photos (Figure 2). This ensured that the inclusion of areas with no plant material along the tomato plant perimeter (cyan ellipse in Figure 2) was minimized, while still including the majority of the plant for assessment. Measurements of PPC were derived following Scarth (2003) that converts vertical digital photos into measurements of PPC, based on the principle described in Van Gardingen et al. (1999).

The physical condition of each plant in the trial was assessed visually on January 7. Senescent plants were labeled as “poor” condition, while plants with no visibly green photosynthetically active leaves and branches were labeled as “dead.” All other green plants were labeled as being in “good” condition. Based on these observations, empirical relationships were assessed between the field data and UAV imagery.

Yield was measured from each of the harvested tomato plants between January 18–26. This was done by manually counting the number of both mature and immature fruits on each plant and weighing them. Fruit maturity was assessed based on color, with immature fruits being green and fruits with some redness characterized as mature. For small plants (<1 kg shoot mass), all fruits > 3 mm in diameter were counted and weighed. For large plants (>1 kg shoot mass), a representative subset of the whole shoot was selected, and all fruits > 3 mm in diameter were counted, weighed and this subset data was used to extrapolate overall yield by multiplying the measured yield by the ratio of the whole shoot mass and the shoot mass of the selected subset. The number of fruits ranged from 1 to 3349 per plant with an average number of 528 fruits/plant. Yield ranged from 0.1 to 1433 g per plant with an average yield of 227 g/plant.

### UAV Image Collection and Pre-processing

UAV-derived RGB imagery was collected using a Zenmuse X3 camera (Dà-Jiāng Innovations, Shenzhen, China) for all of the eight field campaigns, while additional multi-spectral green (530–570 nm), red (640–680 nm), red edge (730–740 nm) and near infrared (NIR) (770–810 nm) imagery were collected with the Parrot Sequoia sensor (Parrot SA, Paris, France) for the last two campaigns. Both cameras were mounted on a DJI Matrice 100 (Dà-Jiāng Innovations, Shenzhen, China) Quadcopter for coincident data capture. All UAV imagery were collected close to solar noon under clear sky conditions at a speed of 2 m/s and a height of 13 m. The Universal Ground Control Station (UgCS) Client application (SPH Engineering, SIA, Riga, Latvia) was used to autonomously collect the multi-spectral imagery with 68% sidelap and 83% along-track overlap, recording photos once every second. The RGB imagery was collected with an 82% sidelap and 78% along-track overlap, recording photos every 3 s. The sidelap was constrained by the field of view of a simultaneous thermal image data capture (not presented in this research). The RGB and multi-spectral imagery was processed in Agisoft PhotoScan (Agisoft LLC, St. Petersburg, Russia) to produce a georeferenced orthomosaic and Digital Surface Model (DSM) for each data capture. A 13 m flying height produced a pixel size of 0.5 and 1.12 cm for the RGB and multi-spectral orthomosaics, respectively. Based on the relationship between the field-derived spectrometer measurements and the digital numbers of the six radiometric calibration panels within the orthomosaics, the digital numbers were converted to at-surface reflectance for the RGB and multi-spectral imagery, using an exponential and linear empirical line correction, respectively (Ahmed et al., 2017; Johansen et al., 2018). This produced coefficients of determination (*R*^2^) > 0.98 for all band combinations. In order to produce a DSM, a dense point cloud was required. The dense point cloud was produced from Structure-from-Motion at “high” quality and using “mild” depth filtering to avoid removing points representing the tomato plant canopies. A Digital Terrain Model (DTM) was produced based on RGB imagery collected for the bare ground prior to planting. A Canopy Height Model (CHM) was produced by subtracting the DTM from the DSM.

### Object-Based Image Analysis for Delineation of Tomato Plants

**FIGURE 3:**
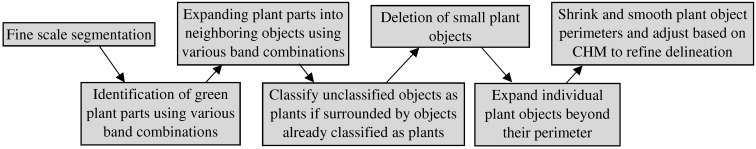
Flowchart showing the individual steps in the object-based image analysis process used for delineating the tomato plants.

**FIGURE 4:**
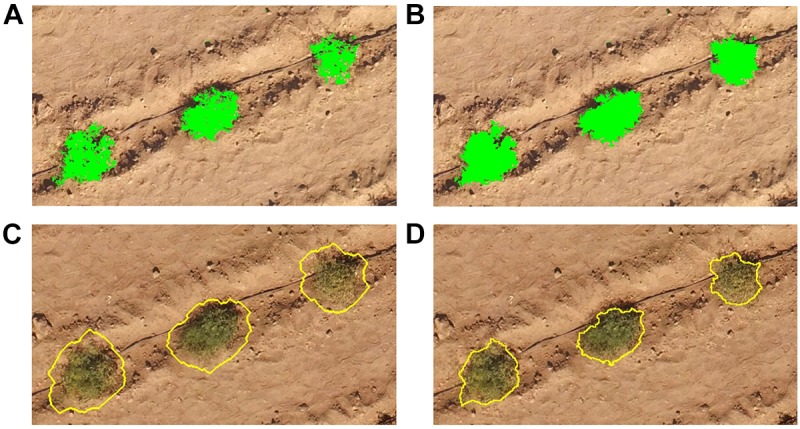
Individual steps in the object-based image analysis used to automatically delineate the tomato plants from the UAV-based RGB and multi-spectral imagery, using **(A,B)** various band combinations and thresholds of these (green objects), and pixel-based object resizing to **(C)** expand and subsequently **(D)** shrink plant objects (yellow outlines).

An object-based approach was developed in the eCognition Developer 9.3 software (Trimble, Munich, Germany) to delineate all tomato plants from each of the eight RGB and two multi-spectral image captures (Figure 3). First, a fine scale segmentation (multiresolution segmentation algorithm, scale factor = 6, shape = 0.1, compactness = 0.5) based on the three visible bands and the Green-Blue index (Table 1) for the RGB imagery, was performed to cluster pixels together with similar spectral information. The multi-spectral bands and all five band combinations reported in Table 1 were used to segment the multi-spectral imagery with the same multiresolution segmentation settings as the RGB imagery. Objects representing the green parts of the tomato plants were identified using empirically defined thresholds for a number of spectral band combinations (see Table 1: Thresholds 1 and Figure 4A). The identified areas were then expanded using a region-growing algorithm to grow into neighboring objects as long as slightly more relaxed thresholds (see Table 1: Thresholds 2) were fulfilled. A restriction imposed on this region-growing was that unclassified objects could only be classified as tomato plants if they bordered objects already classified as tomato plants (Table 1: Thresholds 2 and Figure 4B). This procedure was looped until the threshold conditions were no longer met to ensure all objects belonging to an individual tomato plant were encapsulated into a single large object. Unclassified objects surrounded by tomato plant objects were then classified as, and merged with, the respective tomato plant objects. Objects classified as tomato plants with an area < 150 cm^2^ and occurring more than 30 cm from a larger tomato plant object were labeled as unclassified, as these represented incorrectly classified objects.

**Table 1:** Band combinations and associated thresholds (TH) used for the initial tomato plant identification in the object-based image analysis for the RGB and multi-spectral UAV imagery.

RGB band combinations	TH 1	TH 2	Multi-spectral band combinations	TH 1	TH 2
Red-Green	<22	<40	NDVI: (NIR-Red)/(NIR+Red)	>0.26	
RGB Brightness:	>45	>45	Brightness:	>33	>30
(Red+Green+Blue)/3	<160	<160	NIR+RedEdge+Red+Green		
Green-Blue Vegetation Index: (Green-Blue)/(Green+Blue)	>0.19	>0.19	NIR-RE-NDVI: ([(RedEdge+NIR)/2]-Red)/ ([(RedEdge+Red)/2]+Red)	>0.30	>0.22
Green-Red Vegetation Index: (Green-Red)/(Green+Red)	>–0.06		RE-NDVI: (RedEdge-Red)/(RedEdge+Red)	>–0.23	>0.17
(Red-Green)/Blue	<0.37	<0.7	(NIR+RedEdge)/2	>10	
Relative border to tomato plants		>0.001	Relative border to tomato plants		>0.001


A pixel-based object resizing algorithm was then used to grow the tomato plants classified in the above steps beyond their perimeter, to ensure that all parts of the individual plants were included within the expanded objects (Figure 4C). This object expansion was only permitted into unclassified pixels to prevent neighboring tomato plants from merging. These large objects were then shrunk pixel by pixel in multiple loops, into areas of either bright bare ground or dark shaded ground, while smoothing the object perimeter and preventing shrinking into plant parts of the objects with NDVI values > 0.20 (Figure 4D). Finally, the CHM was used to expand the tomato plants into areas with a CHM value > 15 cm, if these were bordering the delineated tomato plants. Dead plants were identified based purely on the CHM and a height threshold of 15 cm. Each of the UAV-based delineation results were visually assessed and manually edited as necessary. The field-derived measurements of plant dimensions were used for evaluation of the UAV-based delineation results.

### Extraction of Phenotypic Traits

The delineation of individual tomato plants allowed for an assessment of plant area. Plant area calculations were undertaken both at the plot level and at the individual plant level. From each plant object, it was possible to automatically derive a measure of object area, length (longest axis), and width (perpendicular to longest axis). Plant growth rates were assessed using the mapped plant area from the eight UAV campaigns. To calculate growth rate, the change in plant area between individual UAV campaigns was divided by the number of days between the campaigns. This provided seven growth rate measurements per plant through the growing season.

To map plant condition, an empirical relationship was developed between the field observations and derived vegetation indices (Table 1). To do this, 50% of randomly selected observations were used to empirically set NDVI and RE-NDVI values to < 0.35 and < 0.22, respectively, based on the multi-spectral imagery (Robson et al., 2017) to map plants that were either dead or in poor condition. These values were set to minimize errors of commission (incorrect inclusion of an observation, causing overestimation) and omission (incorrect exclusion of an observation, causing underestimation) based on the field assessment of plant condition carried out on January 7. For the RGB imagery, a Green-Red Vegetation Index (Motohka et al., 2010) threshold value was set to -0.19 for discriminating between plants in good and poor/dead condition. Field observations of dead plants and plants in poor condition were treated as one category because of the difficulty of spectrally discriminating these two categories in the imagery. As all image datasets were normalized to at-surface reflectance, the empirically set thresholds were applied to all other image dates to map plants in poor/dead condition.

The average value per plant of a range of vegetation indices (Table 1) were correlated against field-derived measurements of PPC, using linear and quadratic regression analysis, with predictive performance examined based on the calculation of RMSE. The vegetation indices producing best-fit equations with the largest R^2^ value and the lowest RMSE for both the RGB and multi-spectral imagery were used to predict PPC for all other dates. This was deemed appropriate as all image datasets were normalized to at-surface reflectance.

### Identifying the Best-Performing Tomato Plant Accessions

Each of the UAV-based phenotypic traits were first correlated against field-harvested total yield mass (mass of mature and immature tomato fruit) per plant to determine their suitability for predicting the best-performing accessions. Jung et al. (2018) used a sequential procedure for cotton genotype selection based on UAV-derived canopy cover and open boll related phenotypic features. They compared the selected UAV-based entries to the highest yielding entries, with the UAV-selected entries matching 80 and 73% of the minimum and average lint yield, respectively. We adopted a similar approach to Jung et al. (2018) in this research, using a sequential procedure whereby tomato plant accessions were gradually eliminated based on the four UAV-derived phenotypic traits (condition, plant area, growth rate and PPC) to identify the best-performing accessions in relation to yield. Thresholds for this elimination process were set empirically.

To identify the best-performing tomato plant accessions, the time-series information on plant condition was used first. All of the plants that were initially classified as being in good condition, but proceeded to either poor/dead condition or went missing (e.g., due to wind damage or removal if dead), were identified based on the UAV-based condition classification results. When evaluating the UAV-based condition results for each of the 200 accessions (each accession had three replicates in each of the two treatments), those accessions with ≥ 2 (with ≥ 1 of these being salt-treated to incorporate salt tolerance into the condition assessment) of the six plants classified as either poor/dead condition or missing, were omitted from further analysis, as it was deemed undesirable to have only ≤ 4 out of 6 plants surviving the growing season. The remaining accessions were then used for the next elimination stage.

In the next stage, the mean area of the five or six tomato plants per accession for the last UAV data collection on January 14 was used. To establish a threshold based on plant area to identify the best-performing tomato plants, the tomato plants with the top 10% highest yield were identified based on the field data of total yield mass per plant. These 120 tomato plants (out of a total of 6 plants × 200 accessions; i.e., 1200 plants) were then sorted based on UAV-derived plant area to identify the smallest plant area, which was then used as a threshold. Plant accessions with a mean plant area below this threshold on January 14 were eliminated.

For each tomato plant, seven growth rate measurements were then obtained from the eight UAV datasets. The maximum value of these seven measurements was assigned to each plant. The maximum growth rate occurred within a short period around the end of November for the majority of plants. The tomato plants with the top 10% highest field-assessed yield were then selected, and out of these 120 plants, the smallest maximum growth rate value was used as a threshold. The average value for the maximum growth rate of the five or six plants belonging to each accession was then used to omit accessions if the average value fell below this threshold.

PPC was then used for the eight UAV image data captures. PPC values varied throughout the growing season with 25 and 75 quantiles ranging from around 66–86% and 82–91%, respectively. The mean PPC per accession was first calculated for each of the UAV datasets. Then, the number of PPC occurrences over both 85 and 80% were counted for the eight data captures. These thresholds were empirically set based upon the UAV-derived PPC values. Plant accessions with mean PPC values under 80% occurring in at least four out of the eight data captures, were excluded unless at least three out of the eight captures had PPC mean values over 85%. These thresholds were set empirically against the field-derived yield data. The remaining tomato plant accessions that were not eliminated based on their condition, area, growth rate and PPC values, were then compared with the ranked list of yield performance per accession based on field-derived data.

A similar approach was used for assessment of control and salt-treated plants separately. As there were only three plants per accession for each treatment, accessions with two or three plants in poor/dead condition or missing were omitted. Thresholds for plant area, maximum growth rate and PPC were obtained as described above, but only based on 600 plants split between the two treatments.

To gain further information on similarities and differences between the evaluated phenotypic traits of the 200 accessions mapped from the UAV imagery (i.e., condition, area, maximum growth rate, PPC), a principal component analysis was undertaken, using (1) all accessions and all plants (i.e., up to six plants per accession); (2) all accessions and all salt-treated plants (i.e., up to three plants per accession); and (3) all accessions and all control plants (i.e., up to three plants per accession). For the principal component analysis, the average plant area on January 14, average PPC for all eight campaigns, and the average value of the maximum growth rate were derived for each accession. Condition was given a number from 0 and 6 based on the number of plants in good condition on January 14 per accession. For the separate analysis of the salt-treated and control plants, the condition number ranged from 0 and 3, with only three plants per accession.

## Results

### Delineation of Tomato Plants

Object-based image analysis was used to delineate all tomato plants from the 10 UAV image datasets. For the first five campaigns, > 99% of all tomato plants were automatically detected, with 7–12% of the automatically detected plants requiring manual editing to ensure accurate delineation of the perimeter of the tomato plants. The omission rate increased to between 1.7–5.4 and 0.96–2.1% for the last three campaigns for the RGB and multi-spectral imagery, respectively. The increased rate was due to the presence of dead and senescent plants exhibiting reflectance characteristics similar to neighboring bare ground and falling below the 15 cm CHM threshold used to discriminate plants from rocks and other small features above ground level (Table 2). Consequently, this increased the need for manual editing, with 9–16% of plants requiring adjustment for the RGB imagery, respectively. In comparison, adjustments of 5–12% of plants were required for the multi-spectral imagery. The NIR and red edge bands of the multi-spectral imagery facilitated the mapping of senescent plant parts as well as shaded leaves compared to the RGB imagery, explaining the difference in the plants requiring adjustment, although the higher spatial resolution of the RGB imagery improved the ability to map small senescent plants in some cases. For example, the use of the higher spatial resolution RGB imagery enabled identification of three plants that were incorrectly omitted using the multi-spectral imagery of January 7, 2018 (Table 2). However, the RGB image color and texture, i.e., spatial arrangement of color, of senescent plant parts appeared very similar to that of disturbed bare ground and bare ground with scattered shadows from plant branches and leaves. Commission errors also occurred on a few occasions, where weeds were not correctly removed. In such cases, these were manually deleted. When comparing the area of those plants that were manually adjusted to the automatically delineated area prior to adjustment, it was found that ≤ 10% of the plant area was adjusted in 88.7% of cases. Using measured plant length and width for comparison with the automatically delineated plant area, an *R*^2^ value of 0.85 (*n* = 132) with an RMSE of 0.052 m was achieved, with smaller plants slightly overestimated and larger plants slightly underestimated in length.

**Table 2:** Number of plants delineated per plot for the RGB and multi-spectral (MS) imagery for each of the eight campaigns and in brackets, number of plants omitted by the automatic delineation process validated against visual assessment of the RGB and multi-spectral imagery.

Sensor	Date	Control plot 1 no. of plants	Salt plot 1 no. of plants	Salt plot 2 no. of plants	Control plot 2 no. of plants	Total no. of plants
RGB	9 Nov	275 (2)	287 (2)	260 (3)	261 (3)	1083 (10)
RGB	16 Nov	294 (1)	294 (0)	291 (0)	293 (0)	1172 (1)
RGB	23 Nov	295 (0)	294 (0)	291 (0)	294 (0)	1174 (0)
RGB	30 Nov	295 (0)	294 (0)	292 (0)	294 (0)	1175 (0)
RGB	6 Dec	296 (0)	294 (0)	292 (0)	291 (1)	1173 (1)
RGB	20 Dec	294 (6)	291 (7)	291 (5)	289 (2)	1165 (20)
RGB	7 Jan	293 (16)	285 (15)	281 (6)	268 (2)	1127 (39)
MS	7 Jan	290 (8)	285 (9)	281 (5)	268 (2)	1124 (24)
RGB	14 Jan	256 (6)	254 (27)	267 (16)	268 (7)	1045 (56)
MS	14 Jan	256 (5)	255 (4)	267 (4)	268 (1)	1046 (10)


From Table 2 it can be seen that the number of delineated plants increased from November 9–16 as a result of some plants being too small to identify in the first collected UAV dataset after manual editing. The number of plants started decreasing significantly after December 20 as a result of the destructive effects of a number of sandstorms, breaking plant stems and contributing to the death of about 10% of plants prior to harvest. Dead plants were removed prior to harvest to enable their yield to be assessed, and hence some plants were absent in the last two UAV data captures.

### Assessment of Plant Growth

**FIGURE 5:**
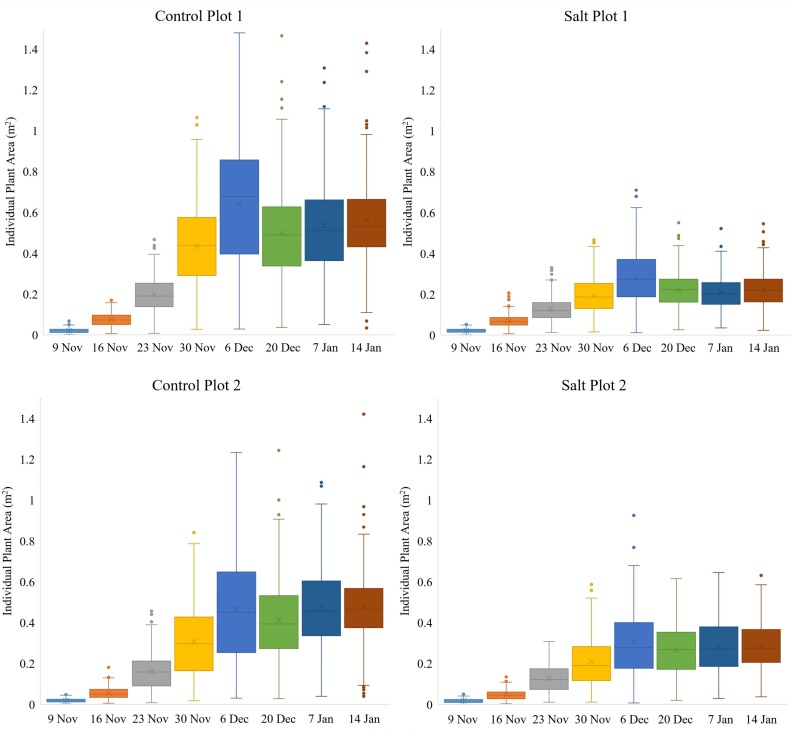
Box-and-whisker plots, showing the variation throughout the growing season in area of individual plants, occurring within each of the four plots. The boxes cover the data from first quartile (Q1) to third quartile (Q3) (interquartile range (IQR)) with the line through each box and the X, displaying the median and mean values, respectively. The whiskers show the limits of Q1–1.5(IQR) and Q3+1.5(IQR) and dots indicate outliers from the population of plants.

The time-series of delineated tomato plants derived from the eight UAV campaigns, allowed an assessment of growth rates on an individual plant and per plot basis. The dominating conditions affecting the phenotypic traits were saline irrigation and high wind speeds associated with sandstorms. As can be seen in Figure 5, the steady increase in plant area per plot over time was evident until December 6, but with a smaller increase for the two salt plots. Saline irrigation was initiated on November 14, and already by the following week, a clear distinction in plant area and daily growth rates could be observed between the plants in the control and salt plots. By December 6, the median plant area for control plots 1 and 2 and salt plots 1 and 2 was 0.68, 0.45, 0.27 and 0.28 m^2^, respectively. Significant sandstorm damage with wind gusts > 15 m/s occurred between December 6–20, resulting in a reduction in plant area per plot. These winds caused branches to break as well as damage to the stems of several plants. Between December 20 and January 7, some recovery and continual growth of plants was observed in the two control plots and salt plot 2, whereas a slight decrease in plant area per plot occurred for salt plot 1. After January 7, plant area per plot became difficult to interpret due to the removal of dead plants. However, on average the remaining plants still continued to grow slightly, even in the two salt plots, until January 14 (Figure 5). At this time, the RGB imagery showed an overall smaller plant area (370.63 m^2^ for all four plots) than that mapped using the multi-spectral imagery (404.52 m^2^ for all four plots): again the result of both senescent and dead parts of the plants being difficult to either automatically or manually delineate. On January 7, the difference in mapped plant area between the RGB and multi-spectral imagery was smaller (412.2 versus 420.57 m^2^, respectively) because of the removal of several dead plants prior to UAV data collection.

**FIGURE 6:**
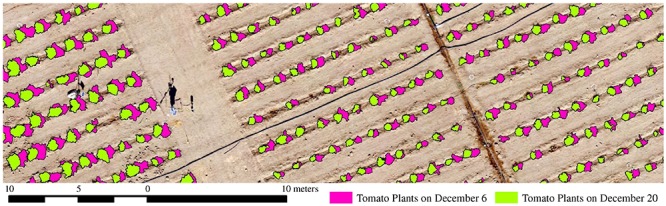
Shifts in tomato plant location due to sandstorms occurring between December 6 and 20, 2017.

While the two control plots had a significantly larger average plant area and daily growth rate than the two salt plots for individual plants from November 9 to December 6 (Figure 5), there were still discernable differences between the two control plots and between the two salt plots. Control plot 1 had the highest growth rate between November 23–30 (0.0336 m^2^/day) and the second highest growth rate between November 30 and December 6 (0.0286 m^2^/day), both of which were higher than the highest growth rate of control plot 2 (0.0228 m^2^/day), which occurred between November 30 and December 6. These differences in growth rates were likely due to differences in environmental conditions between the plots (e.g., exposure to wind). Salt plots 1 and 2 had similar growth rates between November 9 to December 6, with their highest growth rate of 0.0124 and 0.0135 m^2^/day, respectively, occurring between November 30 and December 6. A common characteristic for all four plots was the increase in plant area variability of individual plants from November 9 to December 6 (Figure 5). Between December 6 and 20, salt plot 1 sustained more damage and a reduction in average plant area and growth rate than salt plot 2 due to a strong sandstorm on December 19. With a wind direction from the northeast, salt plot 1 experienced direct exposure, whereas salt plot 2 was sheltered behind control plot 2. Control plot 2, facing northeast, was less impacted by the sandstorm than control plot 1 in terms of average plant area (Figure 5). This was attributed to the larger plant area in control plot 1, with more force behind the movement from side to side of larger plants due to wind gusts. The sandstorm caused a significant decrease in plant area variability (Figure 5) and physical movement of individual plants toward the southwest (Figure 6). From December 20, the growth rates remained low for the remainder of the growing season, with little variation in plant area within the individual plots, and even less variability in the area of individual plants toward January 14 (Figure 5).

### Assessment of Tomato Plant Condition

**FIGURE 7:**
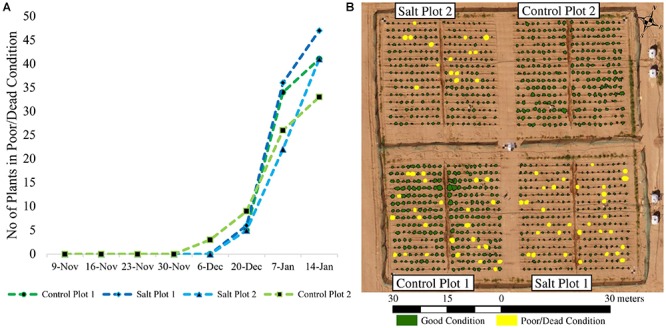
**(A)** Cumulative number of plants either missing or being in a poor/dead condition throughout the growing season; and **(B)** mapped plants in either good or poor/dead condition on January 7, 2018. Missing plants were either blown away during the sandstorms or removed if they were dead to allow their yield to be assessed prior to harvesting the plots. At the time of flight on January 7, all dead plants had been removed in control plot 2.

For the January 7 campaign, 62 plants were visually classified as being either dead or in poor condition, while 56 were missing and 1006 plants were identified as being in good condition (Figure 7). Using 50% of these data for training, empirical thresholds were established from the coincident UAV-based vegetation indices to maximize the number of plants mapped correctly. Based on the Green-Red Vegetation Index (for the RGB imagery) and NDVI and RE-NDVI (for the multi-spectral imagery), 29 out of the 31 plants used for validation were correctly mapped as being in poor/dead condition (omission error = 6.5%). The two plants that were not identified had index values close to the set thresholds. The set thresholds were a compromise to reduce both omission and commission errors. The commission error of the RGB and multi-spectral imagery were 13.9% (5 incorrectly classified plants) and 8.8% (3 incorrectly classified plants), respectively. This was because some plants (RGB: 3, multi-spectral: 3) consisted of dead or senescent plant material, while still having some remaining green leaves, whereas other plants (RGB: 2) were very sparse but spread out, so that the delineated plant area included background reflectance characteristics from the soil, which reduced the index values. A deterioration of plant condition started from December 20 after the first sandstorm. This, combined with additional subsequent sandstorms, resulted in 130 plants missing (removed if dead) and 32 being in poor/dead condition on January 14 (Figure 7).

### Plot and Plant Analysis of Plant Projective Cover

**FIGURE 8:**
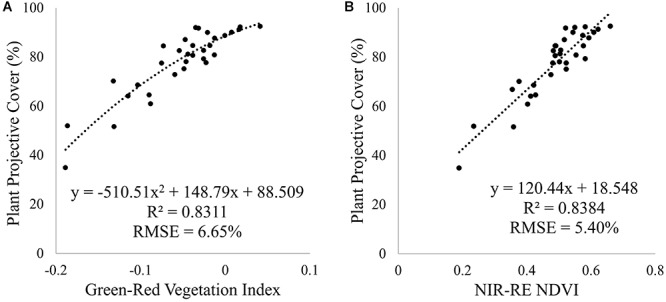
Relationship between field measured PPC and **(A)** the Green-Red Vegetation Index and **(B)** NIR-RE NDVI derived from the UAV RGB and multi-spectral imagery, using a second order polynomial and linear regression, respectively. The best-fit equations were used to predict PPC for all tomato plants. These relationships were based on field and UAV imagery acquired on January 7, 2018.

**FIGURE 9:**
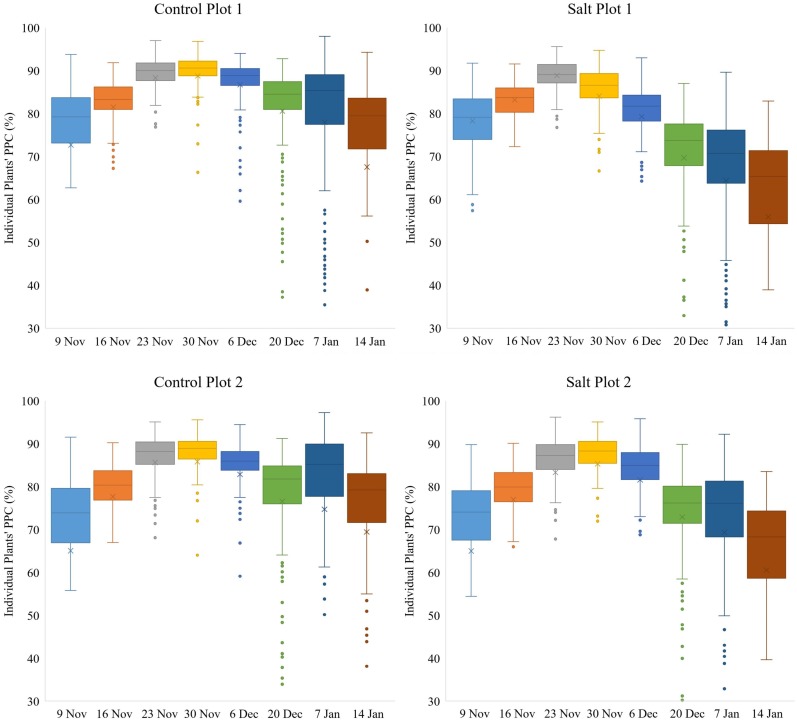
Box-and-whisker plots, showing the variation throughout the growing season in PPC of individual plants, occurring within each of the four plots. The boxes cover the data from Q1 to Q3 with the line through each box and the X, displaying the median and mean values, respectively. The whiskers show the limits of Q1–1.5(IQR) and Q3+1.5(IQR) and dots indicate outliers from the population of plants.

To predict PPC for all tomato plants within the plant trial, a relationship between field measured PPC and a range of vegetation indices was determined for the January 7 campaign. For the multi-spectral imagery, the NIR-RE NDVI (Table 1) produced the best correlation and the lowest RMSE, while the Green-Red Vegetation Index best predicted PPC for the RGB imagery (Figure 8). Employing a second order polynomial prevented overestimation of PPC for plants with Green-Red Vegetation Index values > -0.05 for the RGB imagery. Comparing the predicted PPC values derived from the best-fit equations from the RGB and multi-spectral imagery collected on January 7 yielded similar results (RMSE = 5.31%, *n* = 1124). As all image datasets were normalized to at-surface reflectance, the best-fit equations were applied to all the other datasets to assess PPC variation for the UAV image time-series (Figure 9).

Variation in PPC between plants within the two control plots and salt plot 2 decreased gradually from November 9–30 and from November 9–23 for salt plot 1 (Figure 9), suggesting that the plants with initial low PPC caught up within three weeks, while plants with initial high PPC did not increase as much in terms of the absolute amount of PPC. PPC peaked between November 23–30 for the four plots (Figure 9), which was 1–2 weeks prior to the maximum recorded plant area, as shown in Figure 5. This might have been attributed to compact plants (around November 23) becoming more spread out due to growth of longer branches (around December 6), and hence decreasing the average PPC. Another contributor might have been the occurrence of a shift in plant energy resources away from the transfer of water and nutrient uptake toward producing fruit rather than foliage at the end of November, which may have contributed to the subsequent reduction in PPC per plant (Davies et al., 2000; Kavvadias et al., 2017). Similar to our study, Enciso et al. (2019) also found canopy cover of tomato plants to peak approximately 1 month prior to harvest. The effects of the sandstorms in combination with the two different treatments may have caused the variation in PPC to increase more for the salt plots than the control plots from December 20 to January 14. Similarly, the decrease in PPC between the time of the maximum recorded PPC and January 14 was larger for the salt plots (17.02%) than the control plots (10.42%). The median PPC on January 14 for control plots 1 and 2 and salt plots 1 and 2 was 79.47, 79.24, 65.31 and 68.30%, respectively. Despite the overall larger decrease in PPC for the salt plots toward the time of harvest, Figure 9 shows that some plants in the salt plots maintained high PPC, indicating that some accessions may be more salt tolerant, in terms of their ability to maintain a high PPC, than others.

### Identification of the Best-Performing Plant Accessions

**FIGURE 10:**
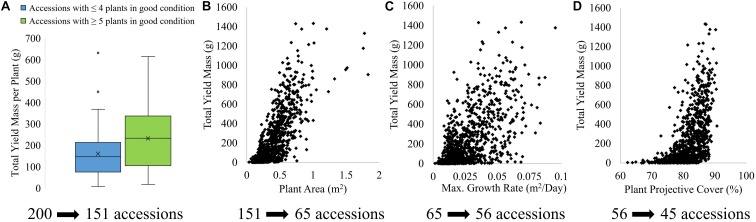
Relationship of field-derived total yield mass per plant with **(A)** plant condition at harvest, **(B)** plant area at harvest, **(C)** maximum daily growth rate throughout growing season and **(D)** PPC of tomato plants at harvest, and the number of accessions eliminated from using each phenotypic traits to identify the best-performing accessions in terms of yield.

The results presented here illustrate just one of many pathways for assessing accession performance. To evaluate the ability to use plant condition, area, growth rate and PPC for prediction of plant performance in terms of yield, the phenotypic traits derived from the UAV imagery were assessed against field-derived total yield mass of tomato fruits (Figure 10). As can be seen in Figure 10A, plant accessions sustaining ≥ 5 plants in good condition on January 14 produced a significantly larger total yield mass (median of 232 g per plant) than those accessions with ≤ 4 plants maintaining good condition (median of 146 g per plant). Figure 10B–D indicate that the total yield mass per plant increased with increasing UAV-derived plant area, maximum growth rate throughout the time-series, and PPC. Hence, the four phenotypic traits presented in Figure 10 were used to eliminate plant accessions to identify the best-performing accessions in relation to yield out of the initial 200 accessions. As three out of the six plants per accession were salt-treated, the sequential elimination approach provided an indication of salt tolerance as well. Figure 10 also shows the number of accessions eliminated at each sequential step, using the four UAV-based phenotypic traits.

Mapped plant condition was used first to eliminate poorly performing accessions, because of the high mapping accuracy of plant condition and the clear distinction between total yield mass per plant based on the number of plants (i.e., ≤ 4 or ≥ 5 plants) in good condition for each accession. Seventy-nine out of the 200 accessions maintained good condition for all six plants. Another 41 accessions had one of the three salt-treated plants either missing (15 accessions) or ascribed as poor/dead condition (26 accessions), while the corresponding three control plants per accession remained in good condition. Thirty-one accessions had either one plant missing (15 accessions) or representing poor/dead condition (16 accessions) in the control plants with all three salt-treated plants being in good condition on January 14. The remaining 49 accessions had two or more plants missing or representing poor/dead condition out of the six plants per accession. These 49 accessions were eliminated from further analysis (Figure 10).

To reduce the remaining 151 accessions further, the tomato plants with the top 10% highest field-assessed yield were selected to determine the average plant area and maximum growth rate per accession below which plant accessions should be eliminated, as described in section 2.7. Based on plant area, a further 86 plant accessions were eliminated. Using maximum growth rate, the number of plant accessions was further reduced from 65 to 56. PPC was used as the final phenotypic trait to further eliminate 11 accessions. As a result of the sequential elimination process, 45 out of the 200 accessions were identified as the “best-performing” in terms of condition, area, maximum growth rate and PPC, which were all related to yield (Figure 10).

Based on the field-derived yield data collected at harvest, it was found that eight out of the top 10 highest yield-producing accessions were identified based on the sequential elimination process, using the four UAV-based phenotypic traits. The two out of the top 10 highest yielding accessions that were incorrectly omitted occurred with two and three plants, respectively, mapped as poor/dead condition on January 14. Despite the high average yield of the three and four remaining plants for the two omitted accessions, it is clearly not desirable if 33 and 50% of plants do not survive the growing season. A total of 14 out of the 20 highest yield-producing accessions were identified, with four out of the six accessions omitted due to poor/dead condition of ≥ 2 plants per accession. The remaining two out of the six accessions were omitted due to either their small plant area or limited growth rate.

A similar approach was used for a separate assessment of the control and salt-treated plant accessions. The sequential elimination process for the control and salt-treated plants identified 36 and 46 out of the 200 accessions, respectively, as the “best-performing.” Among these, the control and salt-treated accessions had 14 in common. However, only five accessions obtained a high ranking in terms of yield for both the control and salt-treated plants, including control accessions ranked as 2, 6, 8, 13 and 32, which corresponded to salt-treated accessions ranked as 11, 1, 23, 13, and 6, respectively, indicating high yield performance for these five accessions under both treatments. Eight and 15 out of the 10 and 20 highest yielding accessions, respectively, were identified for the control plants, whereas nine and 16 of the top 10 and 20 highest yielding accessions, respectively, were detected for the salt-treated plants. These observations demonstrate that phenotypic traits mapped from UAV-based RGB and multi-spectral imagery can be directly applied for selection of accessions for yield optimization.

**FIGURE 11:**
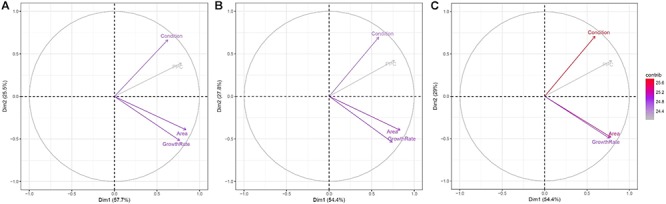
Contribution of plant condition, area, maximum growth rate and PPC for all 200 accessions to principal components 1 (*x*-axis) and 2 (*y*-axis) for **(A)** all plants; **(B)** salt-treated plants; and **(C)** control plants. The values between the brackets indicate the percentage of variance explained by the individual principal components. Each trait’s contribution to the selected principal component is indicated by the length and color of the arrow.

A principal component analysis was performed to obtain information on similarities and difference between the evaluated phenotypic traits for each of the 200 accessions. Principal components 1 and 2 in Figure 11 show similar trends in the contribution of the phenotypic traits toward explaining variance using all plants (Figure 11A), salt-treated plants (Figure 11B) and the control plants (Figure 11C), with principal component 1 explaining between 54.4 and 57.7% and principal component 2 explaining between 25.5 and 29% of the variance. Explaining approximately 83% of the total variance, the first two principal components clearly show the relationship between the variables, with (1) area and growth rate, and (2) condition and PPC being highly correlated. This makes sense as a high growth rate can be assumed to produce a large plant area, and plants in good condition will appear with a denser plant cover. These observations can be directly related to Figure 10, where plant condition and area eliminated 49 and 86 plants, respectively, when identifying the best-performing accessions in relation to yield. Subsequently, the use of maximum growth rate and PPC only eliminated an additional nine and 11 accessions, respectively, because the prior use of the plant condition and area traits had already provided similar information for the elimination process. If focusing purely on the control plants (Figure 11C), plant area and maximum growth rate provide very similar information, whereas for salt-treated plants they are less correlated. Hence, based on the principal component analysis, the UAV-based phenotypic traits to be used for identifying the highest yielding accessions are condition, PPC and either plant area or maximum growth rate, although for salt-treated plants, some additional information is achieved if including all four phenotypic traits.

## Discussion

We sought to address if four phenotypic traits, i.e., condition, plant area, growth rate, and PPC, of tomato plants could be monitored from a time-series of UAV-based RGB and multi-spectral imagery, and if these traits could be used to assess yield performance and salt tolerance in 200 accessions. Eight of the top 10 highest yielding accessions were identified as the best-performing ones in terms of yield. Interestingly, in a related study, Rao et al. (2013) found no correlation between physiological traits (chlorophyll content, leaf sodium content, leaf potassium content, shoot dry mass and plant height) and yield or yield-related traits for *S. pimpinellifolium*. As such, our results present something of a contrast to those in Rao et al. (2013). It is worth noting that their measurements were derived at a single point in time and for individual leaves, which may not be representative of a whole plant, whereas the results presented here were based on the whole plant and their average trait properties, which were assessed eight times during the growing season.

Based on the results from the UAV time-series collected over the growth of the 199 *S. pimpinellifolium* accessions, it seems likely that some of these can be successfully grown in the harsh environments characteristic of Saudi Arabia and the entire Middle East and North Africa region (Pena and Hughes, 2007). Similar studies of wild barley varieties have shown adaptive responses to salt stress in hot dry environments (Ferchichi et al., 2018). This may promote opportunities for enhancing food security in a region with challenging agricultural conditions. In areas that are in short supply of fresh water, any possibility to reduce demands on fresh water by substitution with brackish water can contribute to water and food security efforts. Therefore, identifying accessions that can grow and produce acceptable yield under an irrigation regime of moderately saline water (85–282 mM NaCl or 3,000–10,000 ppm) is of both scientific and practical interest, as they can provide genetic resources to improve commercial varieties. Understanding the mechanisms of plant tolerance to abiotic stressors such as salinity requires identification of the best-performing accessions and their traits. With the use of genomics, the accessions can be sequenced for identification of genetic markers such as single nucleotide polymorphisms (SNP) to genetically map salinity tolerance and the traits contributing to salinity tolerance for introgression into commercial lines (Rao et al., 2012). Such genetic examination is currently being undertaken for the dataset used in this plant trial, with the support of the UAV-based phenotyping presented here.

**FIGURE 12:**
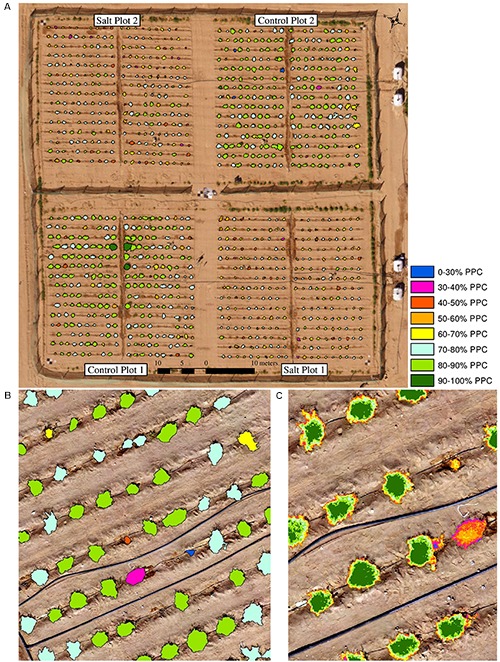
Various reporting scales of PPC based on the RGB imagery collected on January 14, 2018, showing **(A)** PPC for all four plots, **(B)** average PPC of individual plants for an image subset and **(C)** sub-plant PPC distribution of individual plants for an image subset.

Field-based phenotyping is time- and labor-consuming and may lack consistency in multi-temporal data acquisition and for large plant trails (Sugiura et al., 2015; Holman et al., 2016). In this research, it was only possible to field-assess nine plants from each of the four plots in a day. The use of UAV-based monitoring provided a useful tool to scale up the measurements to encompass the entire plant trial of 1200 plants. It also allowed the measurements of phenotypic traits at the plot, plant and even sub-plant level, which is difficult for field-based studies. Figure 12 provides an example of PPC, where statistics can be used to assess all plants in a plot, the average PPC per plant can be derived, and PPC values per pixel within a plant can be used to understand the distribution and condition of foliage. With Structure-from-Motion information derived from multiple UAV-based viewing angles, the 3-dimensional structure of foliage can also be assessed to determine the distribution of biomass and the shape of individual plants (Moeckel et al., 2018; Ziliani et al., 2018). In general, the multi-spectral imagery, including the red edge and NIR bands, was found more feasible than the RGB imagery for mapping traits such as PPC and condition relying directly on spectral information, whereas the RGB data produced near identical results to the multi-spectral imagery for assessment of plant area and growth rates.

While UAV-based phenotyping has demonstrated value for plant trials and agricultural monitoring (e.g., Holman et al., 2016; Burkart et al., 2017; Makanza et al., 2018; Ziliani et al., 2018), there are limitations and assumptions associated with these mapping approaches. The time of the day, resulting in different sun elevation and azimuth angles, may affect mapping results due to changing shadows and bidirectional reflectance distribution function effects (Tu et al., 2018). In this study, we reduced these effects by collecting UAV imagery around solar noon for all eight campaigns. However, seasonal solar elevation angle variations could not be avoided during the growing season. To optimize results and ensure reflectance consistency, all UAV-based datasets were converted to at-surface reflectance using an empirical line correction. However, this approach may also have limitations, as it relies on recorded image digital numbers and their color consistency and constant illumination throughout each flight (Tu et al., 2018). UAV flying height, speed, direction, flight line location, along-track overlap and sidelap were all kept the same for consistency. However, environmental variables such as temperature, humidity and wind speed and direction may introduce data collection variations in an image time-series. The impact of these variables are difficult to quantify and often sensor dependent. This research study provided a near ideal example of UAV-based time-series monitoring of phenotypic traits, as each of the eight campaigns were undertaken with clear sky conditions and low wind speeds, with a sandy soil background, providing a clear contrast to green plant foliage. In addition, all weeds were removed prior to UAV data collection and all plants were hosed down after each sandstorm to avoid reflectance attenuation from sandy leaves. The developed UAV-based approach remains to be tested in more complex and less ideal conditions.

## Conclusion

Plant responses to abiotic stress require systematic testing in field trials to determine desirable phenotypic traits. We presented a novel approach that exploits the use of RGB and multi-spectral UAV image time-series to measure plant area, growth rate, condition and PPC of 199 accessions of the wild tomato species *S. pimpinellifolium* in low salt and high salt-irrigated conditions within an environment with several other significant environmental challenges. The purpose was to use collected data for the selection of the “best-performing” accessions in terms of yield. An object-based image analysis approach to delineate individual tomato plants was found to be useful for this purpose, as our plant level assessment provided significantly better results than previous studies, focusing on field-based leaf-level measurements. Our UAV-based experiment allowed phenotypic assessment of a large number (in this case 1200) of tomato plants on a routine and repeatable basis. The research provides a method to undertake plant trial assessments in a more effective and consistent manner at spatial and temporal scales that, until recently, were not possible to obtain. Our results provide insight into the effects of salt stress on plant area, growth, condition and PPC, of tomato plants, and establish a foundation for further assessment of plant trials at the plot, plant and sub-plant level to facilitate phenotyping and provision of information potentially suitable for plant breeding.

Phenotyping of plants and relating observable traits with yield and salt tolerance can be employed to optimize growth, increase production, promote food security and reduce pressure on freshwater resources. Arid environments such as those found in the Middle East and North Africa, which often face the combined stressors of heat and salinity, are obvious examples of regions requiring specially adapted crops that tolerate high levels of abiotic stress. More generally, improving the productivity of marginal lands and environments is one approach to increasing agricultural production on a global scale: an issue of critical importance to meet the food demands of growing populations. To advance the opportunities that UAVs provide for plant phenotyping studies, further research should focus on deriving additional traits, including biomass, plant height, leaf area index, chlorophyll concentration, and metabolic markers to assess if their inclusion can improve the ability to discriminate the most promising accessions for cultivation. Supplementing the optical imagery used herein with thermal data, or even hyperspectral imagery, is likely to provide additional insights into plant health and function.

## Data Availability

The datasets for this manuscript are not publicly available because they are still under embargo, but a database including field (including all information on the 200 tomato plant accessions) and UAV data will become available in the future. Requests to access the datasets should be directed to KJ, kasper.johansen@kaust.edu.sa.

## Author Contributions

KJ undertook all UAV image processing and analysis, and led the writing of the manuscript, with MFM, MT, and MJM also contributing. MJM designed the plant experiment, and together with GF, SN, and MAM, coordinated activities, including field data collection and the final harvest. YM, BA, SA-M, MZ, and YA were responsible for field equipment and collection of the UAV imagery and field data. MAM led a team of workers to undertake planting, irrigation, fertilization, observation and washing of plants after sandstorms, and harvesting. MT conceived the whole plant experiment and was involved in all aspects of the project. MFM designed the UAV-based experiment, including RGB, multi-spectral, thermal and hyper-spectral data collection, and was involved in all aspects of the project.

## Conflict of Interest Statement

The authors declare that the research was conducted in the absence of any commercial or financial relationships that could be construed as a potential conflict of interest.

## Abbreviations

CHM: Canopy Height Model
DSM: Digital Surface Model
DTM: Digital Terrain Model
IQR: interquartile range
NDVI: Normalized Difference Vegetation Index
NIR: near-infrared, Q1, first quartile
PPC: plant projective cover
Q3: third quartile
RE: red edge
RGB: red-green-blue
RMSE: root mean square error
*S. pimpinellifolium*: *Solanum pimpinellifolium*
UAV: Unmanned Aerial Vehicle
UgCS: Universal Ground Control Software
